# The Homologous Carboxyl-Terminal Domains of Microtubule-Associated Protein 2 and TAU Induce Neuronal Dysfunction and Have Differential Fates in the Evolution of Neurofibrillary Tangles

**DOI:** 10.1371/journal.pone.0089796

**Published:** 2014-02-25

**Authors:** Ce Xie, Tomohiro Miyasaka, Satomi Yoshimura, Hiroyuki Hatsuta, Sawako Yoshina, Eriko Kage-Nakadai, Shohei Mitani, Shigeo Murayama, Yasuo Ihara

**Affiliations:** 1 Department of Neuropathology, Faculty of Life and Medical Sciences, Doshisha University, Kyotanabe-shi, Kyoto, Japan; 2 Department of Physiology, Tokyo Women's Medical University School of Medicine, Shinjuku-ku, Tokyo, Japan; 3 Department of Neuropathology, Tokyo Metropolitan Institute of Gerontology, Itabashi-ku, Tokyo, Japan; Centre Hospitalier de l'Université Laval, Canada

## Abstract

Microtubule-associated protein 2 (MAP2) and Tau are abundant neuronal microtubule-associated proteins. Both proteins have highly homologous carboxyl-terminal sequences that function as microtubule-binding domains. Whereas Tau is widely accepted as a pathoetiological factor in human tauopathies, including Alzheimer's disease (AD), it is not known whether there is a relationship between MAP2 and tauopathy. To better understand the pathological roles of MAP2 and Tau, we compared their behaviors in transgenic *Caenorhabditis elegans* in which MAP2 or Tau was expressed pan-neuronally. Both MAP2 and Tau elicited severe neuronal dysfunction and neuritic abnormalities, despite the absence of detergent-insoluble aggregates in worm neurons. Biochemical analysis revealed that the expressed MAP2 or Tau in worms was highly phosphorylated and did not bind to microtubules. Newly raised antibodies to MAP2 that effectively distinguished between the highly homologous carboxyl-terminal sequences of MAP2 and Tau showed that MAP2 was not involved in the growth process of neurofibrillary tangles in the AD brain. These results indicate that Tau and MAP2 have different fates in the inclusion formation and raise the possibility that MAP2 plays a significant role in neurotoxicity in the AD brain despite the absence of MAP2-aggregates.

## Introduction

Intracellular neurofibrillary tangles (NFTs) are the pathological hallmark of Alzheimer's disease (AD) and other tauopathies, including frontotemporal lobar degeneration, frontotemporal dementia and parkinsonism linked to chromosome 17 (FTDP-17), progressive supranuclear palsy, and corticobasal degeneration [1]. Tau is the principal component of NFTs [2]. Previous studies have indicated that the carboxyl-terminal half of Tau, which includes microtubule-binding domains (MTBDs), makes up the framework of NFTs [3], [4]. By linkage analysis of FTDP-17, more than 30 missense mutations have been found in the exons and introns of the Tau gene. Interestingly, these mutations are almost all localized in or near MTBDs at the carboxyl-terminal region of Tau [1], [5]. These observations strongly suggest an important relationship between the carboxyl-terminal region of Tau and the pathogenesis of tauopathies, although some reports have shown that the amino-terminal region of Tau contributes to its toxicity [6]–[8]. Notably, none of the reports has included a direct comparison in the same animal model system to identify the region responsible for Tau neurotoxicity.

Microtubule-associated protein 2 (MAP2) and Tau differ notably in their subcellular localization within neurons: whereas Tau is distributed abundantly in the axonal compartment, MAP2 is found exclusively in the somatodendritic compartment [9], [10]. Both proteins have the homologous carboxyl-terminal sequences containing the MTBDs and distinct amino-terminal regions (projection domain). The MTBDs comprise three or four imperfect repeats of 31 amino acids in Tau (3R-Tau or 4R-Tau) and typically three repeats in MAP2 [11]. The common physiological role of MAP2 and Tau is considered to be the stabilization of microtubules, which may help maintain neuronal morphology [12], [13]. MAP2 and Tau share highly homologous carboxyl-terminal sequences, but current information about the relationship between MAP2 and tauopathies is far from convincing.

Current data on the expression of human Tau in animal nervous systems have provided a key for better understanding how tauopathies occur. Tauopathy models have been established using mice, nematodes, flies, and fish. *Caenorhabditis elegans* (*C.elegans*), which has a nervous system comprising 302 neurons, is a useful tool for the study of human neurodegenerative diseases [14]–[18]. To investigate the pathological role of Tau, in particular its carboxyl-terminal regions, which are highly homologous with those of MAP2, we produced transgenic worm models and subjected them to behavioral, biochemical, and morphological analyses. In addition, we raised new site-specific antibodies to MAP2 carboxyl-terminal sequences that do not cross-react with Tau. We used these antibodies to investigate the involvement of the homologous carboxyl-terminal regions of Tau and MAP2 in NFT formation in AD brains.

## Materials and Methods

### Ethics statement

Paraffin-embedded human brain sections and frozen autopsy brain tissues were obtained from the The Brain Bank for Aging Research, Tokyo Metropolitan Institute of Gerontology, Japan (URL: http://www.mci.gr.jp/BrainBank/index.cgi). This present study was approved by the ethics committee at Doshisha University and the institute (TMIG).

### Plasmid construction

Human Tau and MAP2 cDNAs were amplified and cloned into *Not*I- and *Bgl*II-digested sites of the pFX vector. The Tau fragments and Tau/MAP2 chimeras were generated by polymerase chain reaction (PCR) and subcloned into the pFX vector. All constructs were under the control of the *unc-119* promoter for pan-neuronal expression, as described previously [19]. Plasmids were verified by DNA sequencing.

### C. elegans strains

*C. elegans* strains were cultured under the conditions described previously [20]. Bristol strain N2 was used as the wild type. Transgenic lines of *C. elegans* were generated as described previously [18], [21], [22]. Briefly, constructs were injected into N2 strains with the marker Pges-1:: EGFP, which creates green fluorescence in the gut. Stable lines were generated by UV irradiation (300 J/m^2^). Integrated lines were backcrossed to N2 twice before use.

To analyze the phenotype for uncoordinated movement (Unc), 20 young adult nematodes were placed on a fresh NGM plate to lay eggs at 20°C for 2 hours, and the adults were removed. Synchronized eggs were cultured at 20°C for 3 days until adults appeared. Twenty new young adult worms per line were transferred individually to new 3.5-cm plates, and Unc was analyzed immediately by multiple researchers under a blind method. The experiments were performed independently at least three times, and at least 60 worms were examined per line. Worms for each line were placed in the SDS sample buffer (0.08 M Tris HCl, 2% SDS, 10% glycerol, 1% 2-mercaptoethanol, pH 6.8), and the mixture was sonicated immediately. The combined lysate of 10 worms per line was subjected to western blotting. The transgenic lines for MAP2c fragments were analyzed without integration. Briefly, young adult worms that showed green fluorescence in gut were picked up and the Unc phenotype was analyzed under the blind method described above. The experiments were performed independently for three times and total thirty animals were analyzed per line.

### Protein extraction and microtubule-binding assay

Four-day-old adult nematodes (tmIs390 for Tau; tmIs849 for MAP2) were harvested in M9 buffer (22 mM KH_2_PO_4_, 22 mM Na_2_HPO_4_, 85 mM NaCl, and 1 mM MgSO_4_) and sonicated in Tris-saline buffer (TS; 50 mM Tris and 150 mM NaCl, pH 7.6) containing protease inhibitors (Roche) and phosphatase inhibitors (1 mM Na_3_VO_4_, 1 mM NaF, 1 µM okadaic acid, and 1 mM β-glycerophosphate). The homogenate was centrifuged at 120,000× *g* for 15 minutes at 2°C. The supernatant was adjusted to a final concentration of 0.5 M NaCl and 2% 2-mercaptoethanol, and was heated to 100°C for 7 minutes. Aggregated proteins and debris were removed by centrifugation at 20,000× *g* for 15 minutes at 2°C. The supernatant was adjusted to 50% ammonium sulfate, kept on ice for 15 minutes, and then centrifuged at 20,000× *g* for 15 minutes at 2°C. The pellet was resuspended and subjected to Lambda protein phosphatase (New England BioLabs, Inc.) treatment for 30 minutes at 30°C. The proteins were purified by repeating the above step of heating and ammonium sulfate precipitation as described above. For the microtubule-binding assay, 4-day-old adult nematodes (tmIs388, mock; tmIs390, Tau transgenic worm; tmIs849, MAP2c transgenic worm) were harvested and homogenized immediately in 0.1 M MES (pH 6.8), 1 mM EGTA, 1 mM MgSO_4_, 2 mM DTT, and 0.5% Triton X-100 containing protease and phosphatase inhibitors at room temperature [23]. The homogenate was centrifuged at 120,000× *g* for 20 minutes at 20°C. The supernatant and pellet were saved separately and subjected to western blotting.

### Preparation of human brain fractions

The frozen autopsy brain tissues from normal control and AD patients were at Braak stage I and Braak stage VI, respectively [24]. Human autopsy tissues from the temporal cortex were sequentially solubilized with TS, followed by TS containing 1% TritonX-100, 1% Sarkosyl, and 1% SDS, as described previously [25]. The Sarkosyl-insoluble, SDS-soluble fractions were subjected to SDS-PAGE followed by western blotting. Total protein was used as a loading control. A PVDF (Polyvinylidene difluoride) membrane was subjected to Lambda phosphatase treatment according to the manufacturer's instructions (New England BioLabs, Inc.) and immunoblotted again. For semiquantitative analysis, Sarkosyl-insoluble, SDS-soluble fractions from normal and AD brain tissues were subjected to western blotting with recombinant Tau (0N4R) or recombinant MAP2c as the standard. Because phosphorylation can affect the immunoreactivity of antibodies, the membranes were treated with phosphatase and then immunoblotted with the anti-Tau (Tau5) and anti-MAP2 (MAP2-#39 and #41) antibodies.

### Immunofluorescence staining

Paraffin-embedded sections (#8097 and #8308) from late-onset AD brains were deparaffinized and pretreated by autoclaving at 120°C for 7 minutes and then with formic acid for 4 minutes. After pretreatment, the sections were incubated with 10% goat serum and then with the primary antibodies. Bound antibodies were visualized with Alexa Fluor 488- or Alexa Fluor 568-conjugated anti-mouse or anti-rabbit IgG antibodies (Invitrogen) and imaged by confocal laser-scanning microscopy (LSM700, Carl Zeiss). The representative results of #8097 are shown.

### *In vitro* aggregation assay

Tau cDNA in the pRK172 vector was a generous gift from Dr. M. Goedert. MAP2c cDNAs were amplified and cloned into *Eco*RI- and *Nde*I-digested sites of the pRK172 vector. Purification of recombinant proteins (Tau 0N4R, Tau 0N3R, and MAP2c) and heparin-induced aggregation were performed according to a previous procedure with some modifications [26]. Briefly, supernatants of *Escherichia coli*-expressed recombinant proteins from BL21(DE3) were applied to a phosphocellulose column and eluted by a gradient of 0.1 M–0.3 M NaCl. The Tau- and MAP2c-containing fractions were purified further by ammonium sulfate precipitation, and the pellets were dissolved in the homogenization buffer containing 0.5 M NaCl and 2% 2-mercaptoethanol. The solution was heated at 100°C for 5 minutes, and the pellet was removed by centrifugation. The resultant supernatant was fractioned by reverse-phase HPLC. For *in vitro* aggregation, 60 µg/ml of heparin was mixed with 10 µM recombinant proteins, 100 mM NaCl, 10 µM thioflavine-T (ThT), and 10 mM HEPES (pH 7.4). The time-dependent changes in ThT fluorescence were measured at 465±35 nm (excitation) and 535±25 nm (emission) for 7 days. After the 7-day incubation, the mixtures were adjusted to 1% Sarkosyl, incubated for 30 minutes at 4°C, and centrifuged at 120,000× *g* for 15 minutes. The obtained supernatants and pellets (Sarkosyl-soluble and insoluble fractions, respectively) were subjected to SDS-PAGE followed by Coomassie brilliant blue staining.

### Antibodies

Site-specific polyclonal antibodies against the carboxyl terminus of MAP2 were raised using KLH-conjugated synthetic peptides (CGGGTPKSAILVPSEK (MAP2-#39), CGGGRVKIESVKL (MAP2-#40), and CGGGITQSPGRSSVAS (MAP2-#41)), which were administered to rabbits via hypodermic injection. We used ELISA to confirm that the raised antisera were specific for recombinant MAP2c and had no cross-reactivity with recombinant Tau. To avoid cross-reactivity with Tau completely, the antisera were passed through Tau-affinity columns in which recombinant Tau was conjugated to the NHS-activated Sepharose 4 Fast Flow (GE Healthcare). These antibodies were then affinity-purified with each corresponding antigen peptide conjugated to the activated thiol Sepharose 4B (GE Healthcare). Purified MAP2 antibodies had no cross-reactivity with Tau detected by western blotting using a gradient dilution of recombinant MAP2c and Tau (Figure S2). Other antibodies that were used are as follows. UNC-119N was raised against a synthetic peptide conjugated to KLH (QQSIAPGSATFPSQMPRGGC). MAP2N (anti-pan-MAP2) was raised against the MAP2 amino-terminal 1–150 amino acids. The antibodies were affinity-purified with the antigenic protein conjugated to activate thiol Sepharose 4B. HM2 (anti-MAP2, Sigma-Aldrich, St. Louis, MO), anti-phospho-MAP2 (Thr1620/1623, Cell Signaling), DM1A (anti-alpha-tubulin, Sigma-Aldrich), HT7 (anti-human Tau, Innogenetics. Zwijndrecht, Belgium), AT8 (anti-phosphoSer-202 and phosphoThr-205 of Tau, Innogenetics), AT100 (anti-phosphoThr-212 and phosphoSer-214 of Tau, Innogenetics), Tau5 (Abnova Corporation), PHF1 (anti-phosphoSer-396 and 404 of Tau, a generous gift from Dr. Davies), and pool 2 (anti-pan-Tau, a generous gift from Dr. Mori) were also used.

### Western blotting

Western blotting was performed as described previously [25]. Briefly, samples were applied to 10% SDS-PAGE and transferred to a PVDF membrane. Bound antibodies were detected by enhanced chemiluminescence (GE Healthcare) or Immunostar LD (Wako Pure Chemical Industries, Ltd., Japan) and imaged using an LAS4000 system (FUJIFILM).

### Statistical analysis

The data are expressed as the mean±SEM and were tested by one-way ANOVA followed by Bonferroni–Dunn *post hoc* test if not mentioned. The significance level is 5%.

## Results

### Neuronal dysfunction is induced by the carboxyl-terminal of Tau

**Figure 1:**
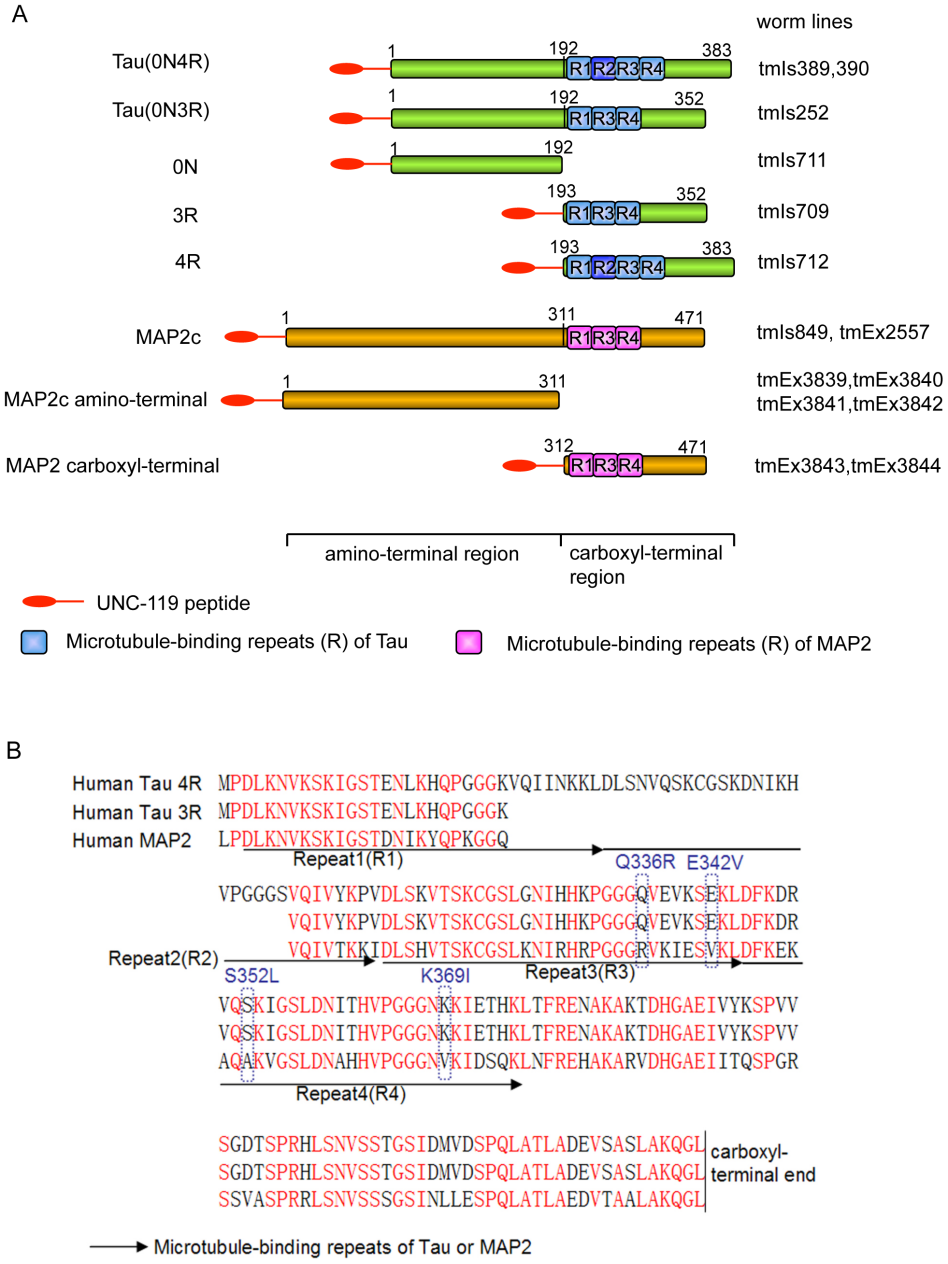
Schematic representation of Tau and MAP2 expressed pan-neuronally in transgenic *C. elegans*. (**A**) Diagrams of expressed proteins, full-length Tau (0N4R and 0N3R), Tau fragments (0N, 3R, and 4R), MAP2c, and MAP2c fragments (MAP2c amino-terminal and carboxyl-terminal) are shown. The Tau and MAP2c microtubule-binding domains (MTBDs) are depicted by blue and purple boxes, respectively. A 23-residue peptide in the UNC-119 amino-terminal assists proper expression and was used as the expression tag linked to the amino-terminus of Tau or MAP2. The numbers written above are the positions of the amino acids. (**B**) Comparison of the carboxyl-terminal amino acid sequences of Tau and MAP2. Identical amino acids are shown in red, and different amino acids are shown in black. Two perfect FTDP-17 mutations, Q336R and E342V, and two imperfect mutations, S325A (S325L in FTDP-17) and K369V (K369I in FTDP-17), that locate within the MAP2 MTBD are indicated.

To identify which region is responsible for Tau neurotoxicity, we developed worm models that expressed full-length 0N4R or 0N3R (the four-repeat 383-residue isoform and three-repeat 352-residue isoform of human Tau, respectively), 0N (corresponding to residues 1–192 of 0N4R Tau), and 4R or 3R (corresponding to the residue of 193– the carboxyl terminus of 0N4R and 0N3R Tau, respectively) driven under the pan-neuronal *unc-119* promoter (Figure 1) [19].

**Figure 2:**
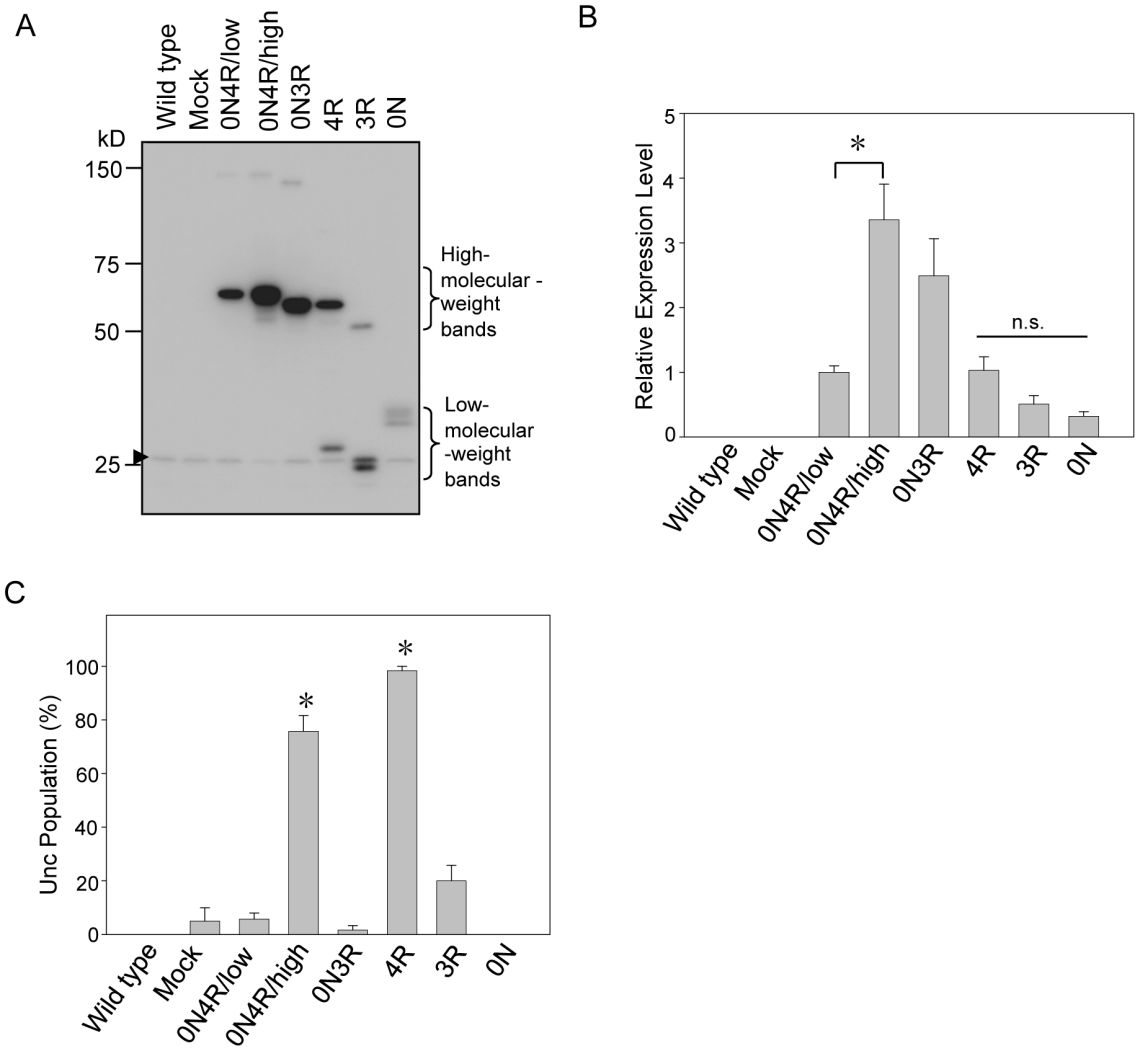
Neuronal dysfunction is induced by the carboxyl-terminal of Tau. (**A**) Representative western blots showing the expressed full-length Tau (0N4R and 0N3R) and its fragments (0N, 3R, and 4R) in transgenic worms. Mock indicates the empty vector-transgenic worm line. Total lysates of the indicated 10 worms were subjected to western blotting using the UNC-119N antibody. The arrowhead indicates endogenous UNC-119. (**B**) The expression of each construct is presented as the relative abundance compared with the 0N4R/low-expressing worm (0N4R/low). n.s. indicates not significant. (**C**) Neurotoxicity was examined in each transgenic worm line. Data indicate the percentage of each worm line that showed uncoordinated movement (Unc). Note that the carboxyl-terminal fragment in 4R-Tg worms had a significantly higher percentage showing the Unc phenotype. 3R-Tg worms showed only a limited degree of Unc, whereas no Unc was observed in 0N-Tg worms. All data are presented as the mean±SEM from three independent experiments. Data were tested by one-way ANOVA followed by the Bonferroni–Dunn *post hoc* test. Asterisks indicate significance versus mock (P<0.005, significance level is 5%).

We first quantified the exogenously expressed proteins in each line (Figures 2A and 2B). There were two independent lines expressing high and low levels of 0N4R Tau. The levels of Tau were 3.5-fold higher in the high-expression line (tmIs390) than in the low-expression line (tmIs389). The expression levels of 0N3R Tau were nonsignificantly lower in tmIs252 than those of 0N4R Tau in tmIs390. Four R and 3R fragments containing MTBDs tend to form high-molecular-mass bands observed above the lower-molecular-mass bands on SDS-PAGE (Figure 2A). The density of total bands, including the high-molecular-mass and the low-molecular-mass bands were quantified together. The expression of Tau fragments did not differ between 4R (tmIs712), 3R (tmIs709), and 0N (tmIs711).

The percentage of the Unc population in each line was quantified as described in the Materials and methods section. High expression of 0N4R Tau led to severe Unc [75.7±5.9%, P<0.0001 versus the mock (tmIs388; the empty vector-transgenic worm line with the expression of UNC-119 peptide tag, 5±5%)] in the worms, but its low expression did not lead to significant abnormalities (5.7±2.3%, P = 0.91 versus mock). 0N3R Tau had lower toxicity than 0N4R Tau at a similar expression level (Figure 2C). To identify the region responsible for Tau-induced neuronal dysfunction, we compared the Unc populations of 4R, 3R, and 0N Tau fragment-expressing worms. As shown in Figure 2C, 4R fragment-expressing worms exhibited the highest percentage with behavioral abnormality (98.3±1.7%, P<0.0001 versus mock). Three R fragment-expressing worms showed a fourfold increase in the percentage expressing the Unc phenotype compared with the mock line, but this increase was not significant (20±5.8% versus 5±5%, P = 0.06). The expression levels of 4R and 3R fragments were similar to those of the 0N4R/low line and 0N3R Tau, and the fragmentation of Tau may enhance its toxicity to neurons. In sharp contrast to MTBD-containing fragments, 0N Tau fragment-expressing worms showed no abnormality. Thus, we concluded that the carboxyl-terminal region of Tau containing MTBDs is essential for its neurotoxicity.

To examine whether over-expression of exogenous proteins cause neuronal dysfunction, GFP was expressed at a level more than threefold higher than that of Tau, but no neuronal dysfunction was observed. In addition to GFP, we also expressed GSK-3beta, Hsp70 or DsRed, any of which did not induce the Unc phenotype observed in the 0N4R/high and 4R fragment-expressing worms (data not shown). These results suggested that in the *C. elegans* system, the Unc phenotype could be induced only by the specific amino-acid sequences other than over-expression itself.

Neuronal dysfunction is also induced by the carboxyl-terminal of MAP2.

Because the 160-residue carboxyl-terminal of MAP2, which contains MTBDs, is highly homologous to that of Tau (see Figure 1B), we asked whether neurotoxicity could also be elicited by MAP2. The low-molecular-mass isoform MAP2c shares its entire sequence with the high-molecular-mass MAP2 isoform and is similar in size to Tau. MAP2c and its fragments-transgenic (Tg) worms were developed using the identical pan-neuronal-expressing system under the *unc-119* promoter, as described above (Figure 1A).

**Figure 3:**
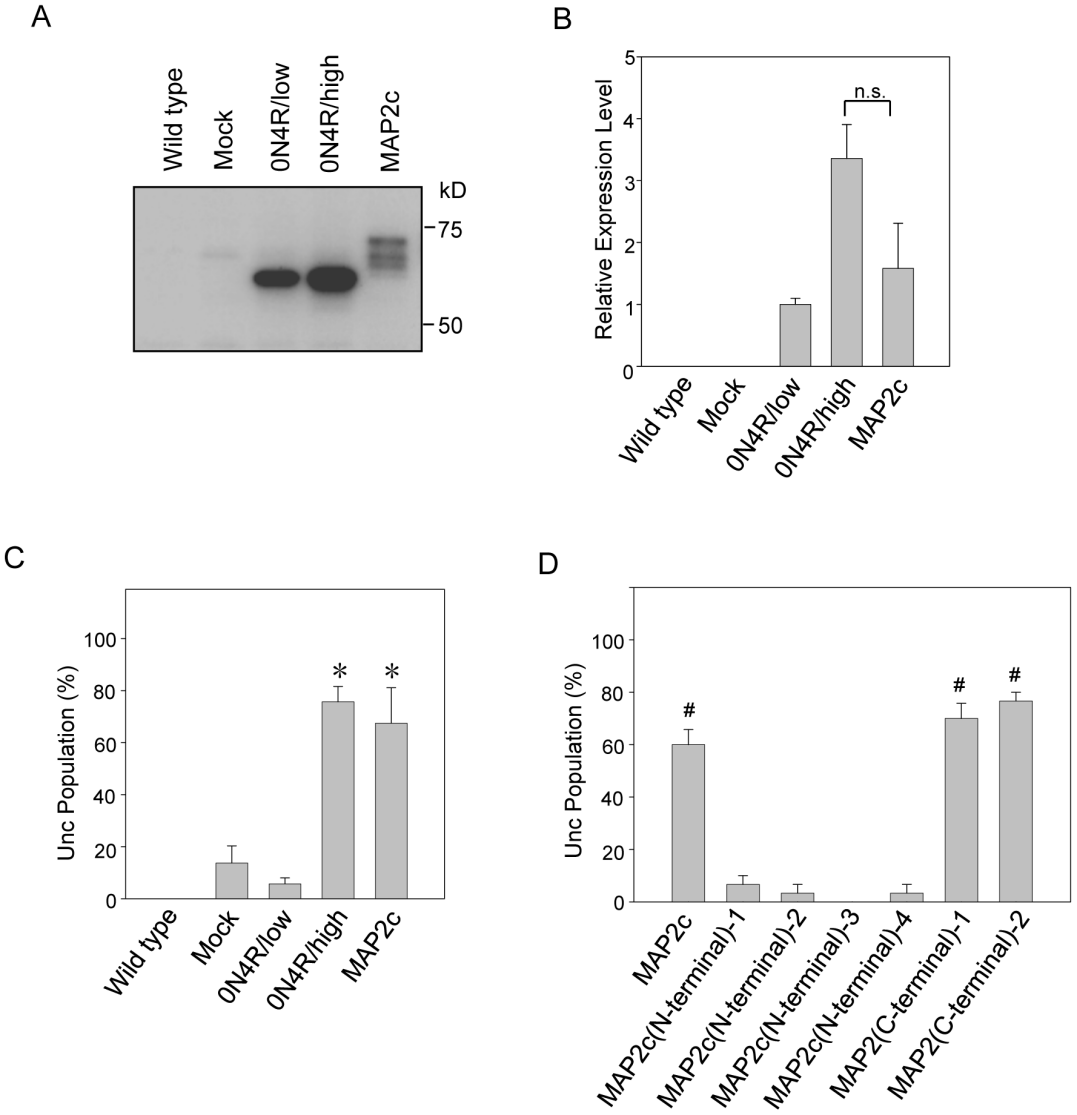
Toxicity of both Tau and MAP2 in worm neurons. (**A**) Representative western blot showing the expression of Tau and MAP2c in transgenic *C. elegans*, as probed with UNC-119N antibody. (**B**) The expression of each construct is presented as the relative abundance compared with the 0N4R/low-expressing line (0N4R/low). No significant difference was observed between the MAP2c and 0N4R/high lines. (**C**) Neuronal dysfunction is induced by MAP2c. Both MAP2c- and Tau 0N4R/high-expressing worms show a significantly higher percentage of worms with the Unc phenotype compared with the mock, indicating that MAP2c was as neurotoxic as Tau. (**D**) The carboxyl-terminal domain of MAP2 is responsible for its neurotoxicity. Unc analysis was performed using the MAP2c full-length (tmEx2557), MAP2c amino(N)-terminal (1 to 4 corresponded to tmEx3839,3840,3841 and 3842, respectively) and MAP2 carboxyl(C)-terminal (1 and 2 corresponded to tmEx3843 and 3844, respectively) Tg lines. The data are expressed as the mean±SEM from at least three independent experiments and were tested by one-way ANOVA followed by the Bonferroni–Dunn *post hoc* test. Asterisks indicate significance versus mock (P<0.005, significance level is 5%). # indicate significance versus MAP2c amino-terminal Tg lines (P<0.005, significance level is 5%).

As shown in Figures 3A and 3B, MAP2c was expressed at lower levels than was Tau in the 0N4R/high line, although this difference was not significant (3.4±0.5-fold versus 1.6±0.7-fold, P = 0.008). The percentage of MAP2c-expressing worms showing the Unc phenotype was similar to that of the 0N4R/high line (67.5±13.6% versus 75.7±5.9%) and both were significantly higher than that of the mock line (Figure 3C), suggesting that MAP2c induces neuronal dysfunction in *C. elegans*. To indentify the region responsible for MAP2c-induced neuronal dysfunction, we compared the Unc phenotype populations of MAP2c amino-terminal, carboxyl-terminal and full-length-Tg worm lines. As shown in Figure 3D, MAP2 carboxyl-terminal fragments-Tg lines as well as the full-length-Tg line showed high percentage of Unc phenotype. In contrast, MAP2c amino-terminal fragments expressing worms showed less behavioral abnormalities. These results indicate that the carboxyl-terminal region of MAP2 is responsible for its neurotoxicity.

### Tau and MAP2 are phosphorylated and do not bind to microtubules in Tg worms

**Figure 4:**
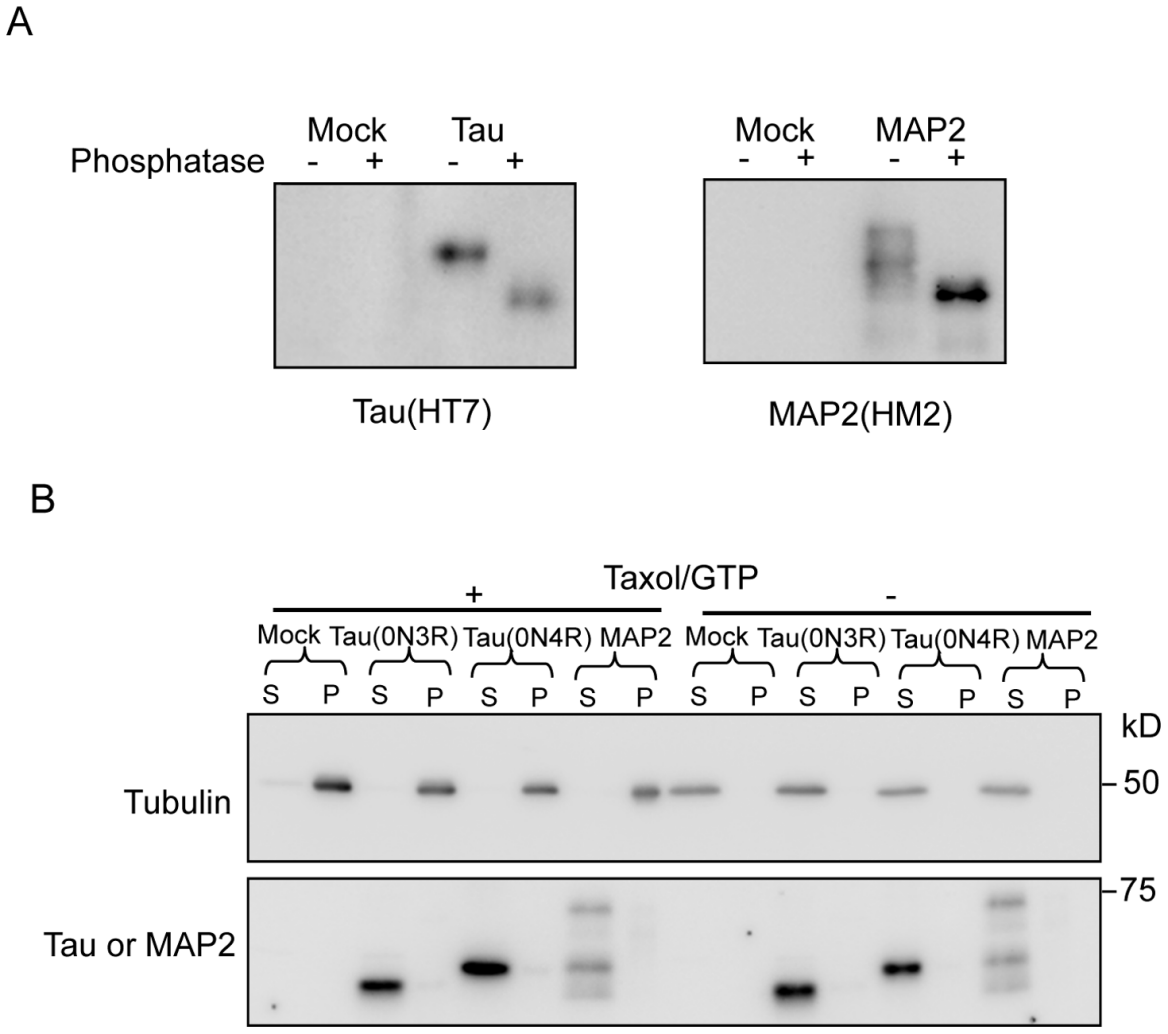
Biochemical characterizations of MAP2c and Tau expressed in transgenic *C. elegans*. (**A**) Both MAP2c and Tau were highly phosphorylated in worm neurons. MAP2c and Tau (0N4R) were purified from the corresponding transgenic worms (MAP2c from tmIs849; 0N4R from tmIs390). Purified proteins were treated with or without phosphatase and subjected to western blotting using the HT7 (anti-human Tau monoclonal) and HM2 (anti-MAP2 monoclonal). (**B**) MAP2c and Tau did not bind to microtubules. The microtubules prepared were stabilized with taxol and GTP, and fractionated into the pellet (P) and supernatant (S). Both MAP2 and Tau remained in the supernatant (S). DM1A (anti-α-tubulin) and anti-UNC-119N (Tau and MAP2c) antibodies were used.

Hyperphosphorylation, which dissociates Tau from microtubules, is a characteristic sign of the AD brain [27], [28]. To examine the phosphorylation and microtubule-binding activity of exogenous MAPs in worm neurons, Tau and MAP2 were purified from transgenic worms and subjected to phosphatase treatment, as described in the Materials and methods section. The phosphatase treatment caused significant mobility shifts of Tau and MAP2 on SDS-PAGE, suggesting that both molecules were highly phosphorylated in the worm (Figure 4A). In addition, Tau (both 0N3R and 0N4R) or MAP2, which were expressed in Tg worms, were recognized respectively by anti-phospho-Tau antibodies (AT8 and AT100) or anti-phospho-MAP2 (Thr1620/1623) antibody (data not shown).

Tau and MAP2-Tg worms were subjected to the microtubule-binding assay. After centrifugation under the conditions in which microtubules were stabilized in the buffer containing taxol and GTP, both Tau and MAP2 purified from Tg worms were recovered in the microtubule-unbound fraction in the supernatant but not in the precipitate, suggesting that they were not bound to microtubules because of abnormal hyperphosphorylation (Figure 4B). As described in the previous study, despite PHF-tau and fetal tau are hyperphosphorylated and share several phosphorylated epitopes, fetal tau can bind to microtubules, but PHF-tau loses the function of microtubules binding [27]. Because Tau and MAP2 were not bound to microtubules in the transgenic worms, the present data suggested that both Tau and MAP2 took abnormal forms in the transgenic worm neurons. The liberation of Tau and MAP2 from microtubule may be necessary for the gain of toxic function. Notably, the solubility of both Tau and MAP2 suggested that their neurotoxicity is mediated through a TritonX-100 soluble-form-dependent mechanism in this *C. elegans* system (Figure 4B).

### Neuritic abnormalities in Tau and MAP2-Tg worms are age dependent

**Figure 5:**
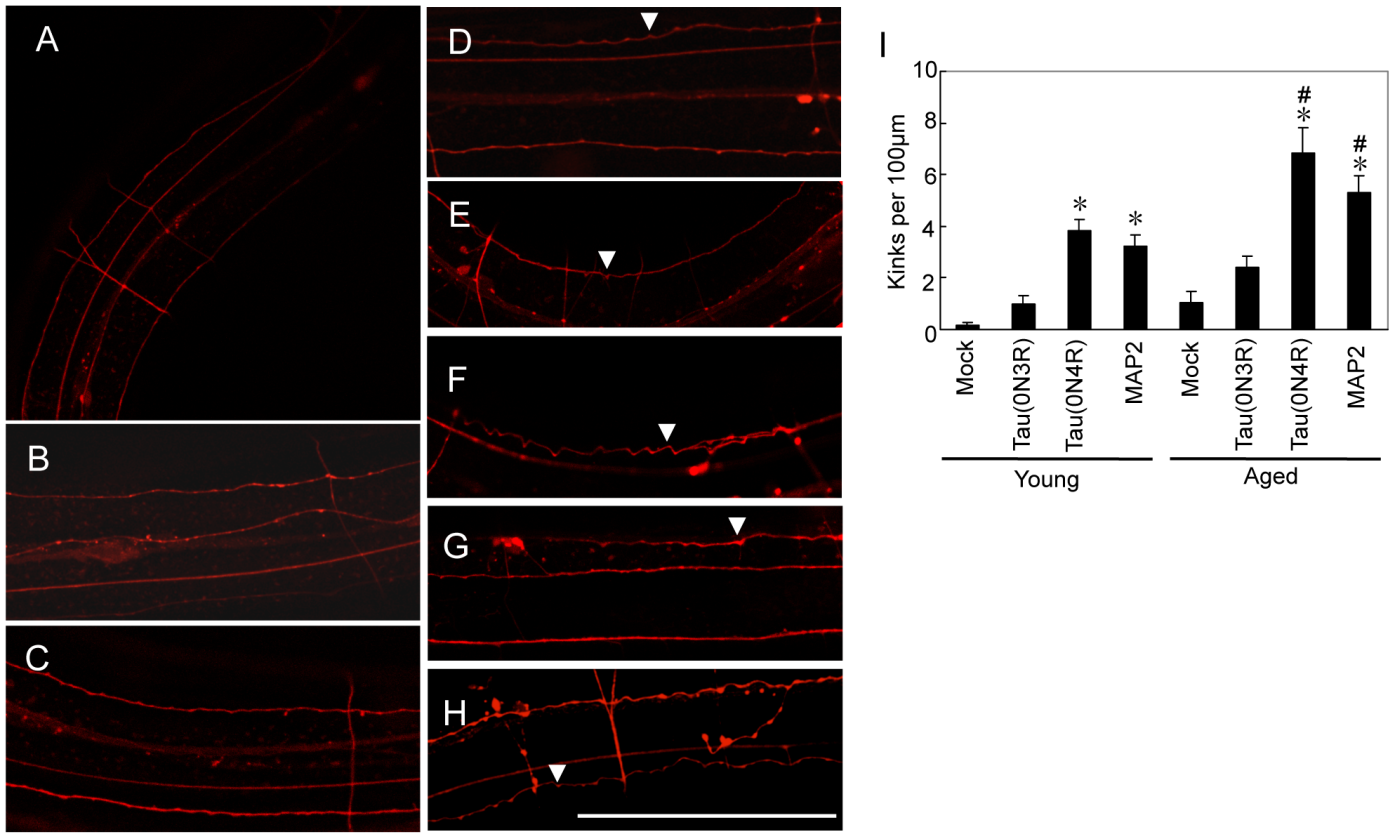
Age-dependent neuritic abnormalities in Tau- or MAP2-expressing worms. (**A–E**) CLSM images of neurites in the posterior part of the worm are shown. (**A**) Mock/DsRed-transgenic (Tg) worm, young. (**B**) Mock/DsRed-Tg worm, aged. (**C**) Tau(0N3R)/DsRed-Tg worm, young. (**D**) Tau(0N3R)/DsRed-Tg worm, aged. (**E**) Tau(0N4R)/DsRed-Tg worm, young. (**F**) Tau(0N4R)/DsRed-Tg worm, aged. (**G**) MAP2/DsRed-Tg worm, young. (**H**) MAP2/DsRed-Tg worm, aged. The scale bar is 100 µm. (**I**) Numbers of abnormal kinks (arrows) per 100 µm neurite. “Young” indicates 4–5 days after hatching, and “aged” indicates 10–11 days. The data are expressed as the mean±SEM. Asterisks indicate significant differences versus mock in each age group (one-way ANOVA followed by Bonferroni–Dunn *post hoc* test). # indicates a significant difference in the young versus aged group in the same line (P<0.05, Student's t-test). n = 21 to 23.

The expression of Tau or MAP2 in neurons induced significant neuronal dysfunction in worms. We hypothesized that this neuronal dysfunction would correlate with morphological abnormalities in these Tg worms. To address this issue, DsRed, a red fluorescent protein, was expressed under a pan-neuronal *unc-119* promoter to visualize living neurons. DsRed-expressing worms were crossed with mock, Tau (0N3R and 0N4R), and MAP2c-Tg worms. Mock/DsRed-Tg (mock line and DsRed double-Tg) worms had relatively straight neurites, which are considered normal (Figure 5A). By contrast, Tau(0N4R)/DsRed-Tg (0N4R and DsRed double-Tg) and MAP2/DsRed-Tg (MAP2c and DsRed double-Tg) worms exhibited obviously abnormal neurites: many kinks were observed along the neurites, which fluoresced red (Figures 5E–5H). Tau (0N3R)-Tg line showed abnormal morphologies to some extent, but not significantly different from the mock line (Figure5). The number of kinks increased from a few per 100 µm in young worms (4–5 days) to several per 100 µm in aged worms (10–11 days), indicating an age-dependent progression in the appearance of these neuritic abnormalities (Figure 5F). To explore whether Tau(0N4R) or MAP2 was expressed in these abnormal neurites, paraffin sections of mock/DsRed-Tg, Tau(0N4R)/DsRed-Tg, and MAP2/DsRed-Tg worms were prepared. Tau and MAP2 were labeled with anti-Tau (pool 2) and anti-MAP2 (anti-MAP2N) antibodies, respectively. Tau and MAP2 were expressed in abnormal neurites having kinks (Figure S1), indicating that MAP2 and Tau induced similar neuronal abnormalities. We note that the distinct subcellular localization of Tau or MAP2 was not observed in the Tg worms.

### MAP2 is not involved in the evolution of NFTs in the AD brain

Next, we examined the contributions of MAP2 and Tau to NFT formation in human AD brains. Following NFT formation, the amino-terminal half of deposited Tau is gradually removed and processed by unknown proteases or spontaneous cleavage through cyclic succinimidyl intermediates, and finally, the residual portion containing MTBDs is left at the PHF core [29], [30]. Thus, only antibodies against the MTBDs or their periphery can detect a full range of old to newly deposited Tau [25]. Because the amino-terminal region of MAP2 is much longer than that of Tau expressed in the adult brain, only antibodies against the MTBDs of MAP2 can resolve precisely whether MAP2 is deposited in NFTs.

**Figure 6:**
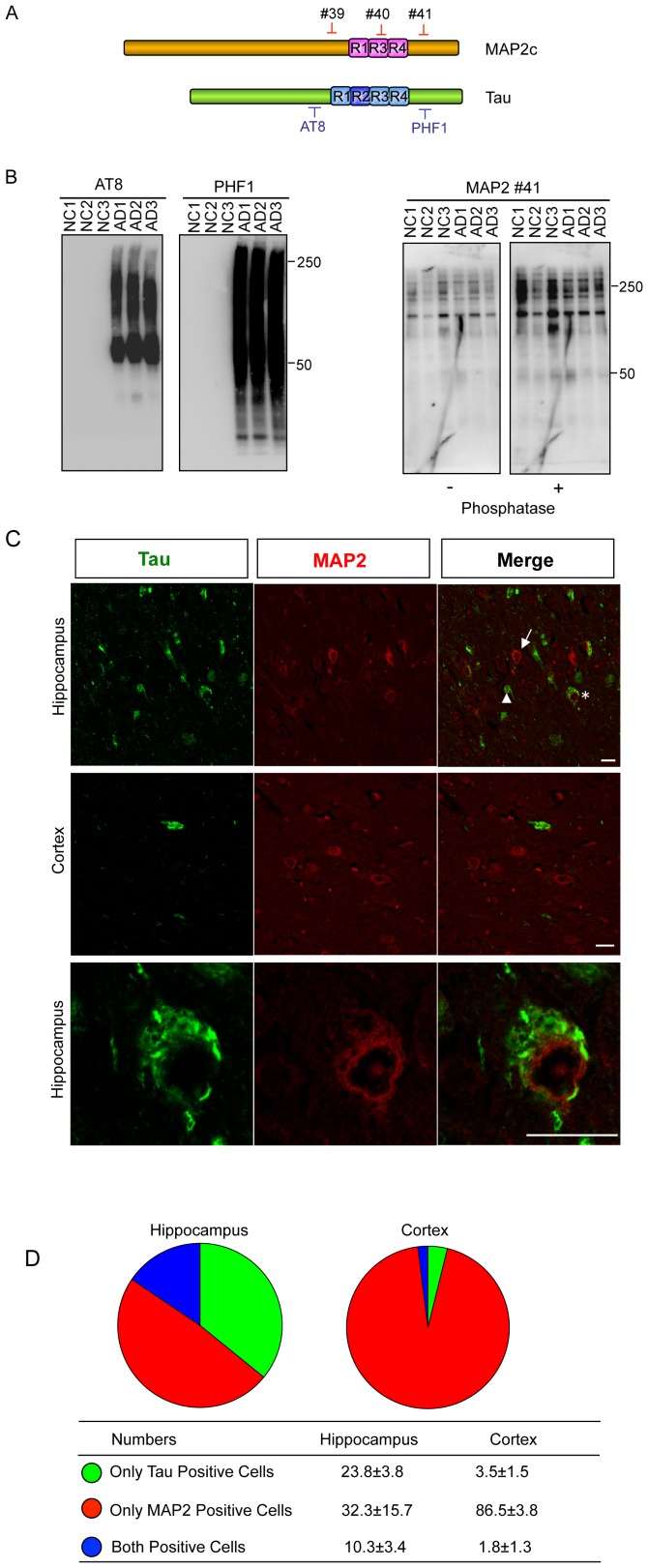
MAP2 is not involved in the growth process of NFTs in the AD brain. (**A**) Diagram of three site-specific anti-MAP2 antibodies and anti-Tau antibodies. (**B**) Human temporal cortex tissues from three AD patients and three normal controls were homogenized sequentially in detergent-containing buffers. Sarkosyl-insoluble, SDS-soluble fractions were prepared and subjected to SDS-PAGE followed by western blotting using anti-Tau antibodies (AT8 and PHF1) and three newly raised site-specific anti-MAP2 antibodies (MAP2-#39 and #40 are not shown). Total protein was used as the loading control. The staining intensity of Tau was increased markedly in the Sarkosyl-insoluble, SDS-soluble fractions from AD brains compared with normal brains. By contrast, the MAP2 antibodies failed to detect any increased patterns in AD brains compared with normal brains. Phosphatase treatment was performed to avoid effects from MAP2 phosphorylation. NC, normal brains; AD, Alzheimer's disease brains. Information about the cases is provided in Table S1. (**C**) Double immunofluorescence staining of the homologous carboxyl-terminal sequences of Tau and MAP2 in the AD brain. AD brain paraffin-embedded sections were double-labeled by anti-Tau antibody (PHF1) and anti-MAP2 antibody (MAP2-#41). Tau but not MAP2 localized in NFTs as well as in NTs. Representative Tau-positive-only neurons (arrowhead), MAP2-positive-only neurons (arrow) and Tau/MAP2-double-positive neurons (star) are indicated. Scale bars = 25 µm. (**D**) Average number of the three neuron types was counted per 640 µm^2^. The data are presented as the mean±SD.

We found that it was very difficult to distinguish MAP2 from Tau using the carboxyl-terminal antibodies because of high homology in the amino acid sequences between the two molecules (see Figure 1). The commercially available anti-MAP2 antibodies whose epitopes are near the MTBDs easily cross-reacted with Tau (data not shown). Thus, we raised three new independent site-specific anti-MAP2 polyclonal antibodies with epitopes that localize to the carboxyl-terminal sequence, either in or near the MTBDs (Figure 6A). Purified anti-MAP2 antibodies did not cross-react with Tau (Figure S2). Several previous trials tried to investigate whether MAP2 is a component of NFTs by using conventional immunochemical approaches, but the results were conflicting [31]–[34]. The confusion may have been caused by the relatively low specificities of the antibodies used [35]. As far as we know, this is the first study of the involvement of the carboxyl-terminal region of MAP2 in NFT formation determined using well-characterized antibodies that showed no cross-reaction with Tau. This distinguishes our immunostaining study from the previous works.

Because acquisition of Sarkosyl insolubility is a significant characteristic of deposited Tau in tauopathies [36], [37], we first examined the Sarkosyl insolubility of MAP2 in the brains of AD patients and normal controls (Table S1). Brain tissues were homogenized sequentially with TS buffer containing Triton X-100, Sarkosyl, or SDS. The Sarkosyl-insoluble, SDS-soluble fractions were immunoblotted with anti-MAP2 and anti-Tau antibodies. As expected, anti-Tau antibodies showed a characteristic smear staining in the fractions prepared from AD brains compared with those from normal brains. However, none of the three site-specific MAP2 antibodies (MAP2-#39, #40, and #41) showed any increase in smear staining in AD samples compared with normal brains (MAP2-#39 and #40 are not shown, Figure 6B). We semiquantified the amounts of Tau and MAP2 in the Sarkosyl-insoluble, SDS-soluble fractions from AD brains and found that the amount of MAP2 in this fraction was far less than that of Tau (Figure S3).

We next examined the involvement of the carboxyl-terminal epitopes of Tau and MAP2 in NFT formation by immunohistochemical staining of paraffin-embedded sections from AD brain. As shown in Figure 6C, NFTs and neuropil threads (NTs) were labeled intensely with PHF1 antibody but not with MAP2-#41(MAP2-#39 and #40 showed similar results, data not shown.), which reacts with the epitope corresponding to that of PHF1 (see Figure 6A). The phosphatase treatment of the section before staining did not change the results. (data not shown). Double staining with Tau and MAP2 carboxyl-terminal antibodies distinguishes three types of neurons in AD brain: Tau-positive-only, MAP2-positive-only, and Tau and MAP2 double-positive neurons. Notably, even in the double-positive neurons, no co-localization of Tau and MAP2 was observed (Figure 6C). Regardless of the brain area examined, of the total number of Tau-positive neurons, about 30–40% were MAP2-positive, suggesting that MAP2 is lost during progression of NFT formation (Figure 6D). Thus, despite the high degree of homology between the carboxyl-terminal sequences of MAP2 and Tau (see Figure 1), they have differential fates the course of in NFT formation; that is, MAP2 does not deposit aggressively like Tau and is lost from NFT-forming cells in the AD brain.

### Tau but not MAP2c forms ThT-positive, insoluble aggregates in the presence of heparin

**Figure 7:**
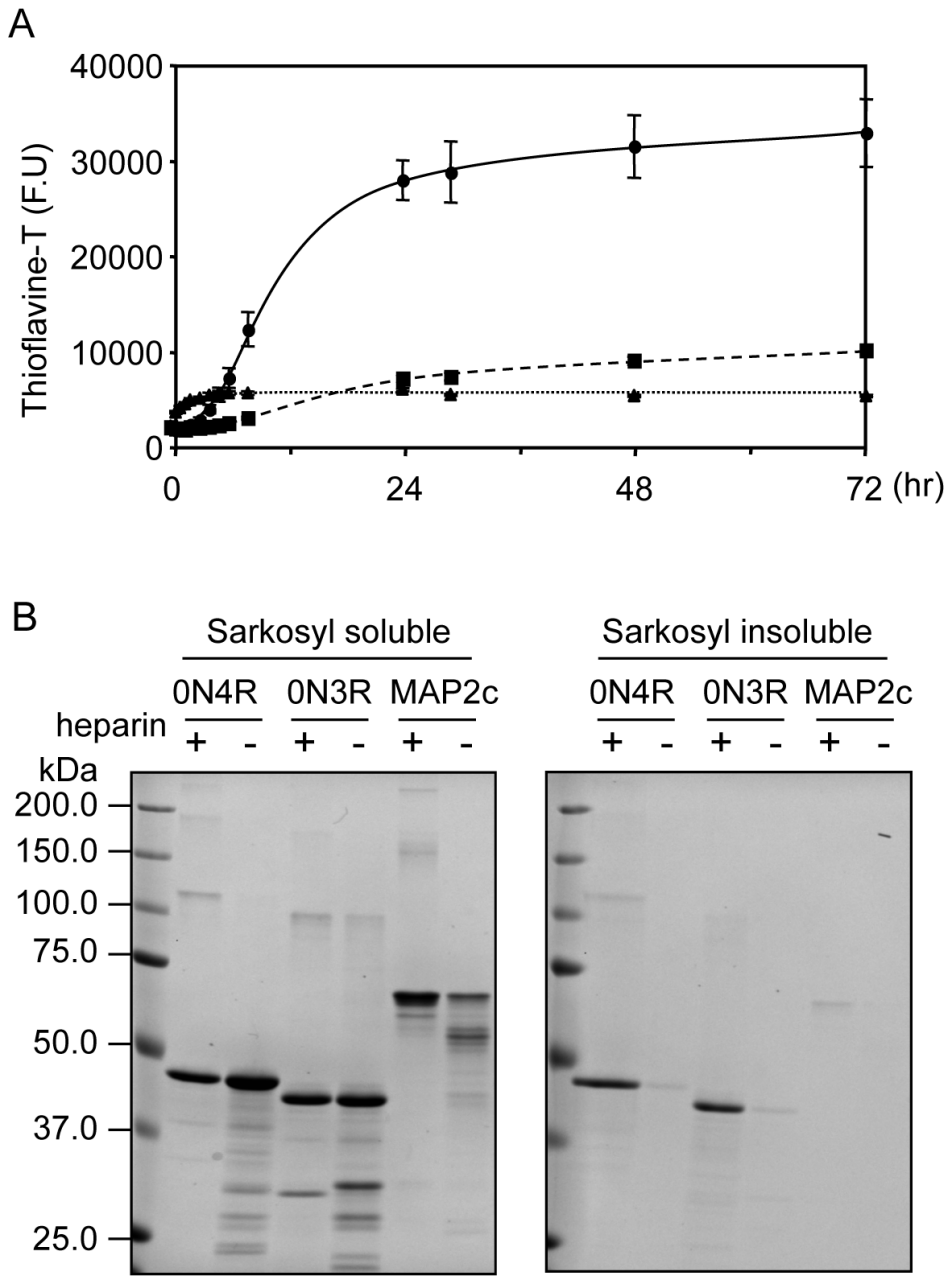
Tau but not MAP2c forms ThT-positive insoluble aggregates induced by heparin. (**A**) The ThT fluorescence of Tau 0N4R isoform (circles), Tau 0N3R isoform (squares), and MAP2c (triangles) aggregates were measured at the times indicated. (**B**) After the 7-day incubation, the amount of Sarkosyl-insoluble proteins in the indicated samples was analyzed by SDS-PAGE followed by Coomassie brilliant blue staining.

We next asked what caused the differential fates of the homologous carboxyl-terminal sequences of Tau and MAP2 in NFT formation. To investigate this dissociation, we used purified recombinant proteins and compared the aggregation potential of Tau and MAP2 in the presence of heparin *in vitro*. ThT fluorescence was used to monitor the aggregation of Tau or MAP2c [26]. As shown in Figure 7A, Tau and MAP2c showed significantly different aggregation profiles. The ThT fluorescence level of Tau remained constant in the first 4 hours and then increased sharply to reach a plateau at about 24 hours. By contrast, the ThT fluorescence level of MAP2c increased more rapidly in the first 4 hours but did not continue and remained constant after 4 hours, suggesting that MAP2c was unable to form large aggregates to the same extent as Tau. This characteristic was not affected by the different amino-terminal halves (data not shown). After incubation for 1 week, the Sarkosyl solubility of the fractions was examined. More Tau than MAP2c was found in the Sarkosyl-insoluble fraction, suggesting that more stable aggregates were formed by Tau than by MAP2c, an observation that is consistent with the results of ThT fluorescence (Figure 7). We acknowledge that the conditions are more complex *in vivo*, but the intrinsic difficulty of MAP2 accumulation might explain its loss from the growing process of NFTs.

## Discussion

In this study, MAP2 or Tau from worm neurons was found to be soluble, strongly suggesting that the neurotoxicity induced by either protein was independent of detergent-insoluble aggregates (Figure 4). This result is consistent with our previous studies suggesting that abnormalities of neurons can be induced by Tau through a nonaggregation mechanism [18]. Santacruz et al. reported NFT-independent neurotoxicity of Tau in a transgenic mice model [38]. This view is also strengthened by a previous quantitative study, which showed that the number of lost neurons was several times higher than NFT-bearing neurons in the AD cortex [39]. These observations suggest that an inclusion-independent mechanism is involved in neuronal loss and thus in the pathogenesis of AD. Here, we found using *C. elegans* that MAP2 and Tau showed similar neurotoxicity in the absence of detergent-insoluble aggregates. Thus, one should not exclude the potential pathological role of MAP2 in the pathogenesis of AD and tauopathies simply because MAP2 barely accumulates in NFTs or in neurons. The highly homologous sequences shared by Tau and MAP2 that exhibit similar neurotoxicity have different fates in the process of NFT formation in the AD brain, as shown by the newly raised MAP2 carboxyl-terminal site-specific antibodies used in this study.

The present results indicated that four-repeat Tau is significantly neurotoxic but three-repeat Tau is not (Figure 2). Because MAP2 is predominantly in the three-repeat form in mammalian neurons, the question arises why three-repeat MAP2 showed significantly greater neurotoxicity than three-repeat Tau. We compared the carboxyl-terminal amino acid sequences, as shown in Figure 1B, which are highly homologous between MAP2 and Tau. Aside from the homologous amino acid sequences, there are some differences in amino acids between Tau and MAP2. It is possible that these distinct amino acids make MAP2 more toxic than three-repeat Tau. The hereditary FTDP-17 form of dementia is caused by mutations in the Tau gene. Previous studies have indicated that these mutations increase Tau pathology through apparently different mechanisms, such as increasing four-repeat Tau isoforms by affecting the splicing pattern or increasing Tau aggregation. Interestingly, two perfect FTDP-17 mutations, Q336R and E342V, and two imperfect mutations, S325A (S325L in FTDP-17) and K369V (K369I in FTDP-17), are observed in MAP2 MTBDs [40]–[43] (Figure 1B). The presence of FTDP-17 mutations in the MAP2 sequence may increase its neurotoxicity to a greater extent.

The present study raises the possibility of a pathoetiological role of MAP2 in the generation of AD and tauopathies. The regions responsible for both aggregation and toxicity are located in the same carboxyl-terminal portions of Tau and MAP2. Thus far, aggregation has been the focus of research, and therefore Tau has been considered exclusively as a pathogenic molecule. Our present study indicates that Tau and MAP2 exhibit toxicity without forming aggregates in worms. This suggests that the involvement of MAP2 in the pathogenesis of AD cannot be excluded because of the absence of its aggregates.

## Supporting Information

Figure S1 Abnormal neurites expressing Tau or MAP2. The paraffin sections of 5-day-old worms (Is388/592, DsRed/mock-transgenic(Tg) worm; Is390/592, DsRed/Tau-Tg worm; Is849/592, DsRed/MAP2-Tg worm) used in Figure 5 were colabeled with anti-DsRed and either pool 2 (anti-Tau) or MAP2N (anti-MAP2). Arrows indicate the normal neurites. Abnormal kinks (arrowheads) are observed in the neurites expressing MAP2 or Tau. Scale bar = 20 µm. n = 6–12.
(TIF)

Figure S2 The three independent site-specific MAP2 antibodies did not cross-react with Tau. Purified recombinant MAP2c and Tau (0N4R) linked with His-tags at the amino-terminals were subjected to western blotting using anti-His tag and anti-MAP2 antibodies (#39, #40, and #41).
(TIF)

Figure S3 Semiquantification of Tau and MAP2 in Sarkosyl-insoluble, SDS-soluble fractions from human autopsy samples from normal and advanced AD brains. Note that standards made of recombinant Tau and MAP2 showed similar staining levels. The amount of Tau was greater than that of MAP2 in the Sarkosyl-insoluble/SDS-soluble fractions from advanced AD brains. NC, normal brain; AD, Alzheimer's disease brain.
(TIF)

Table S1 Information of cases used in this study.
(PDF)

Text S1 Supporting Methods.
(PDF)
