# Supplementary figures and images for: The Homologous Carboxyl-Terminal Domains of Microtubule-Associated Protein 2 and TAU Induce Neuronal Dysfunction and Have Differential Fates in the Evolution of Neurofibrillary Tangles
Source: PLoS One. 2014 Feb 25;9(2):e89796. doi: 10.1371/journal.pone.0089796 (PMC3934940; doi:10.1371/journal.pone.0089796)

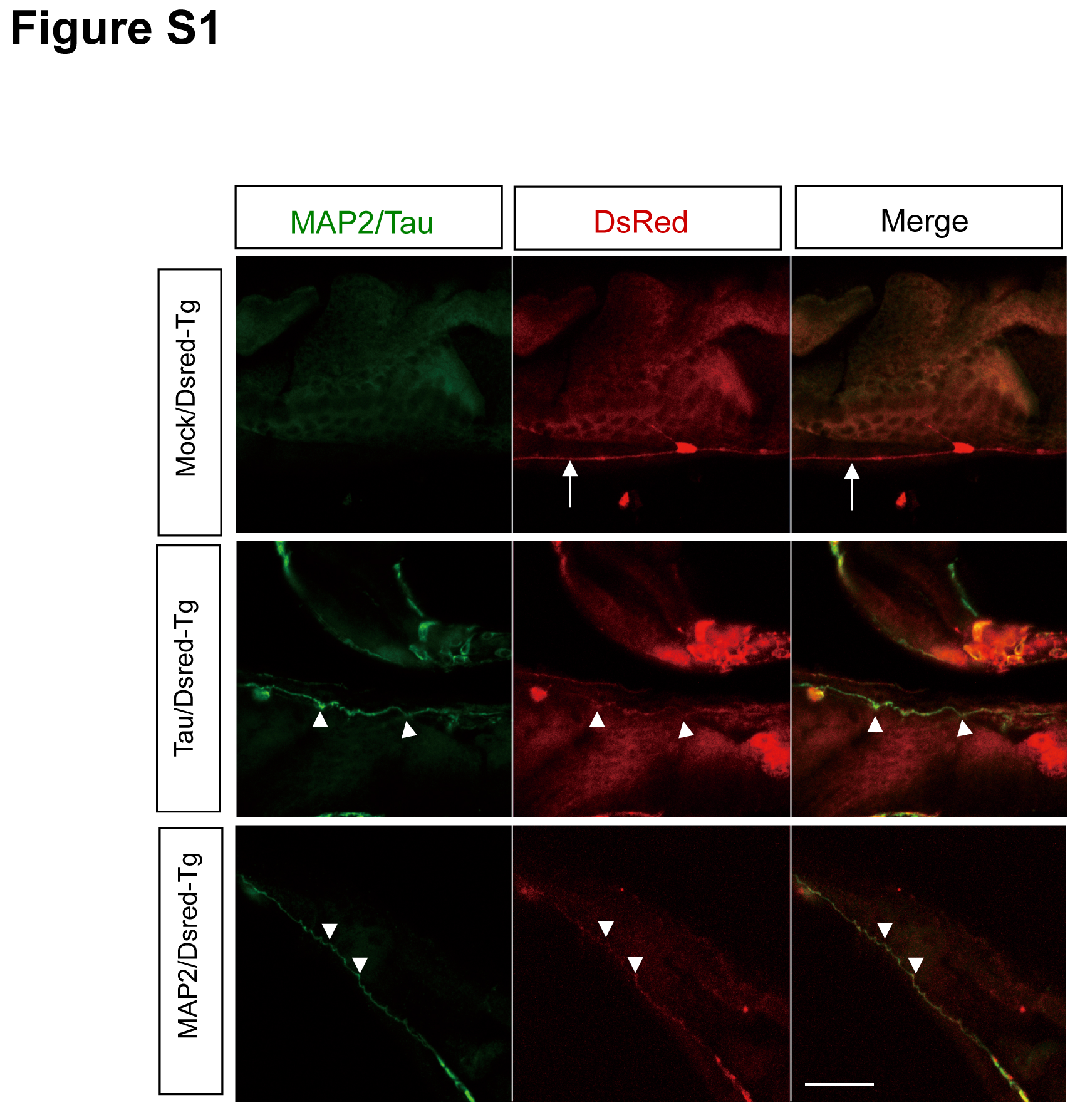

Supplement: Figure S1 — Abnormal neurites expressing Tau or MAP2. The paraffin sections of 5-day-old worms (Is388/592, DsRed/mock-transgenic(Tg) worm; Is390/592, DsRed/Tau-Tg worm; Is849/592, DsRed/MAP2-Tg worm) used in Figure 5 were colabeled with anti-DsRed and either pool 2 (anti-Tau) or MAP2N (anti-MAP2). Arrows indicate the normal neurites. Abnormal kinks (arrowheads) are observed in the neurites expressing MAP2 or Tau. Scale bar = 20 µm. n = 6–12. (TIF) [file pone.0089796.s001.tif]

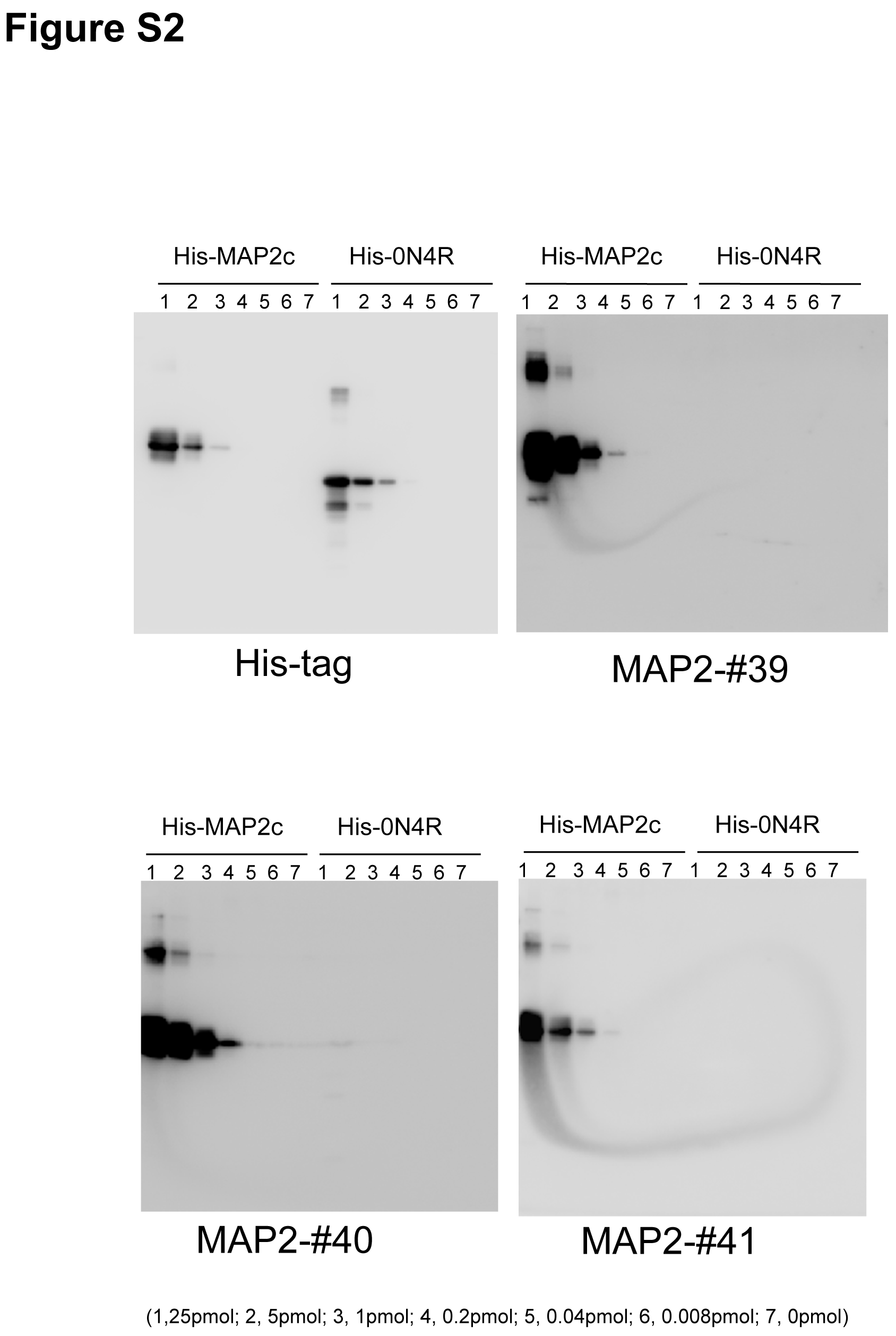

Supplement: Figure S2 — The three independent site-specific MAP2 antibodies did not cross-react with Tau. Purified recombinant MAP2c and Tau (0N4R) linked with His-tags at the amino-terminals were subjected to western blotting using anti-His tag and anti-MAP2 antibodies (#39, #40, and #41). (TIF) [file pone.0089796.s002.tif]

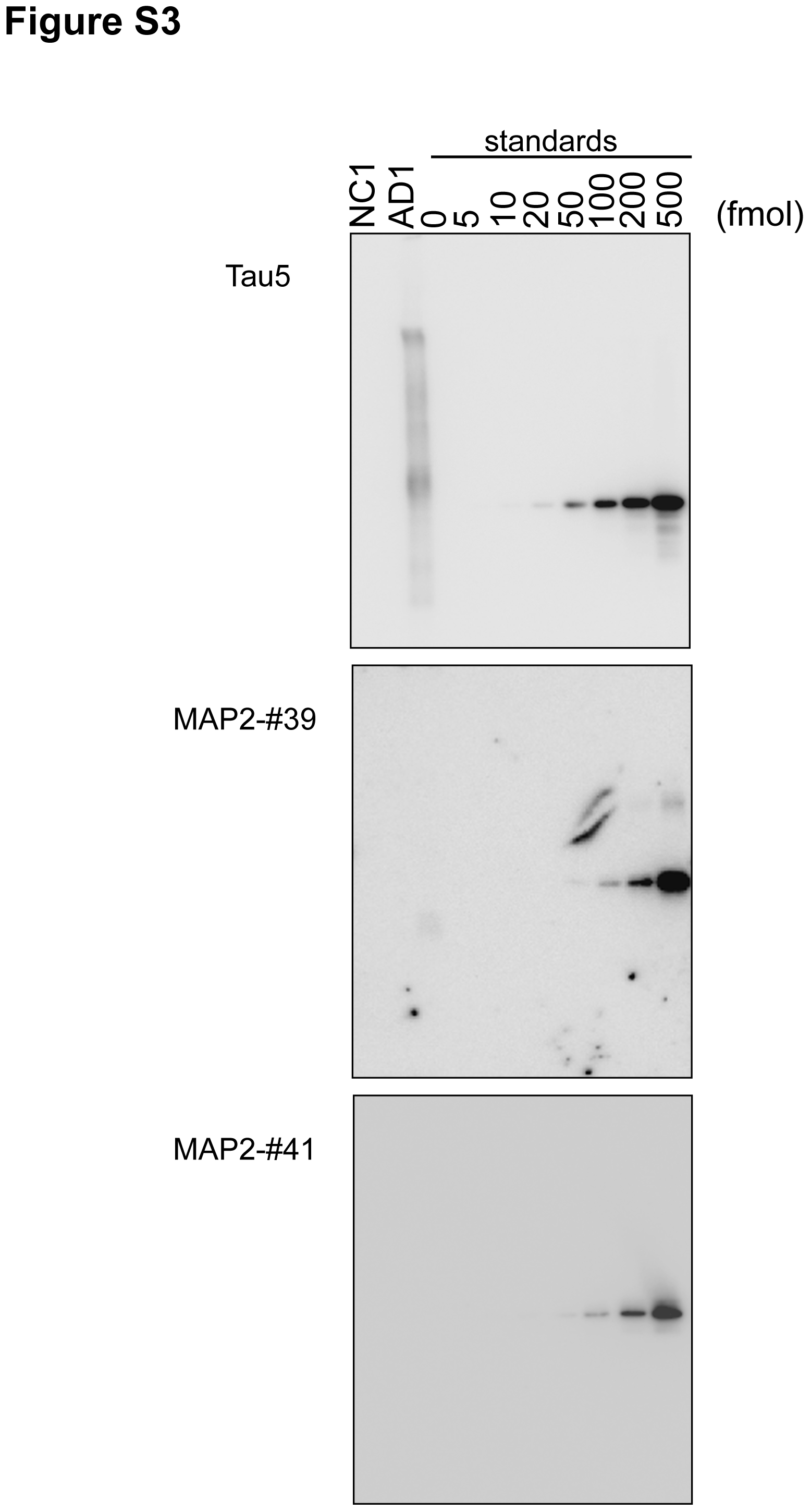

Supplement: Figure S3 — Semiquantification of Tau and MAP2 in Sarkosyl-insoluble, SDS-soluble fractions from human autopsy samples from normal and advanced AD brains. Note that standards made of recombinant Tau and MAP2 showed similar staining levels. The amount of Tau was greater than that of MAP2 in the Sarkosyl-insoluble/SDS-soluble fractions from advanced AD brains. NC, normal brain; AD, Alzheimer's disease brain. (TIF) [file pone.0089796.s003.tif]
