# Supplementary material for: The Homologous Carboxyl-Terminal Domains of Microtubule-Associated Protein 2 and TAU Induce Neuronal Dysfunction and Have Differential Fates in the Evolution of Neurofibrillary Tangles
Source: PLoS One. 2014 Feb 25;9(2):e89796. doi: 10.1371/journal.pone.0089796 (PMC3934940; doi:10.1371/journal.pone.0089796)
Supplement: Table S1 — Information of cases used in this study. (PDF) [file pone.0089796.s004.pdf]

Table S1. Information of cases used in this study.

Case No.		Age/sex	PMI (hours)	Amyloid stage	NFT stage	Tissue
8995	NC1	77/M	10:05	Braak 0	Braak Ⅰ	temporal cortex
8806	NC2	78/M	11:30	Braak 0	Braak Ⅰ	temporal cortex
8847	NC3	82/M	12:16	Braak A	Braak Ⅰ	temporal cortex
8473	AD1	81/M	6:00	Braak C	Braak Ⅵ	temporal cortex
8930	AD2	75/M	9:54	Braak C	Braak Ⅵ	temporal cortex
8687	AD3	83/M	7:02	Braak C	Braak Ⅵ	temporal cortex

Abbreviations: number (No.), male (M), female (F), normal (NC), postmortem interval (PMI).
