# Supplementary material for: The Homologous Carboxyl-Terminal Domains of Microtubule-Associated Protein 2 and TAU Induce Neuronal Dysfunction and Have Differential Fates in the Evolution of Neurofibrillary Tangles
Source: PLoS One. 2014 Feb 25;9(2):e89796. doi: 10.1371/journal.pone.0089796 (PMC3934940; doi:10.1371/journal.pone.0089796)
Supplement: Text S1 — Supporting Methods. (PDF) [file pone.0089796.s005.pdf]

Supporting materials and methods

Immunofluorescence

Five-day-old worms were harvested, washed with M9 buffer, fixed with 4% paraformaldehyde (PFA) at room temperature for 1 hour, and then dehydrated through graded ethanol from 70% to 100%. After treatment with xylene, worms were embedded in paraffin. Paraffin-embedded sections were prepared using a microtome and placed on MAS-coated slide glasses. The sections were deparaffinized, dipped in DAKO buffer for 10 minutes at a temperature above 95°C, washed with TS buffer, incubated with 10% goat serum in TS and with primary antibodies diluted in 1% BSA in TS buffer at 20°C for 16 hours. Bound antibodies were visualized with Alexa Fluor 488- or Alexa Fluor 568-conjugated anti-mouse or anti-rabbit IgG antibodies (Invitrogen) and imaged by confocal laser-scanning microscopy (LSM700, Carl Zeiss).

Expression and purification of recombinant proteins

Human 0N4R Tau and MAP2c cDNA were amplified by PCR with six histidine-coding residues in the forward primers and cloned into the *Nde*I- and *Eco*RI-digested sites of the pRK172 vector. All plasmids were verified by DNA sequencing. The plasmids were transformed into *E. coli* BL21(DE3) competent cells, and expression was induced with 0.5 mM isopropyl-1-thio-β-D-galactopyranoside. The induced proteins were harvested and purified with a His GraviTrap affinity column (GE Healthcare) according to the manufacturer's instructions.
